# COVID-19 in the WHO African region: using risk assessment to inform decisions on public health and social measures

**DOI:** 10.1017/S0950268821001126

**Published:** 2021-05-10

**Authors:** Benido Impouma, Franck Mboussou, Caitlin M. Wolfe, Bridget Farham, George Sie Williams, Opeayo Ogundiran, Roland Ngom, Milse Nzingou, Antoine Flahault, Cláudia Torres Codeço, Ambrose Talisuna, Zabulon Yoti, Olivia Keiser

**Affiliations:** 1 World Health Organization, Regional Office for Africa, Brazzaville, Congo; 2 Institute of Global Health, University of Geneva, Geneva, Switzerland; 3 College of Public Health, University of South Florida, Tampa, Florida, USA; 4 Fundação Oswaldo Cruz, Rio de Janeiro, Brazil

**Keywords:** COVID-19, risk of spread, WHO African region

## Abstract

Successive waves of COVID-19 transmission have led to exponential increases in new infections globally. In this study, we have applied a decision-making tool to assess the risk of continuing transmission to inform decisions on tailored public health and social measures (PHSM) using data on cases and deaths reported by Member States to the WHO Regional Office for Africa as of 31 December 2020. Transmission classification and health system capacity were used to assess the risk level of each country to guide implementation and adjustments to PHSM. Two countries out of 46 assessed met the criteria for sporadic transmission, one for clusters of cases, and 43 (93.5%) for community transmission (CT) including three with uncontrolled disease incidence (Eswatini, Namibia and South Africa). Health system response's capacities were assessed as adequate in two countries (4.3%), moderate in 13 countries (28.3%) and limited in 31 countries (64.4%). The risk level, calculated as a combination of transmission classification and health system response's capacities, was assessed at level 0 in one country (2.1%), level 1 in two countries (4.3%), level 2 in 11 countries (23.9%) and level 3 in 32 (69.6%) countries. The scale of severity ranged from 0 to 4, with 0 the lowest. CT coupled with limited response capacity resulted in a level 3 risk assessment in most countries. Countries at level 3 should be considered as priority focus for additional assistance, in order to prevent the risk rising to level 4, which may necessitate enforcing hard and costly lockdown measures. The large number of countries at level 3 indicates the need for an effective risk management system to be used as a basis for adjusting PHSM at national and sub-national levels.

## Introduction

The novel coronavirus, severe acute respiratory syndrome coronavirus 2 (SARS-CoV-2), first identified in Wuhan City, Hubei Province, China in December 2019, has caused a global outbreak of the disease COVID-19 [1], formally declared a pandemic by the World Health Organization (WHO) on 11 March 2020 [2].

The WHO, the United Nations Agency lead for directing and coordinating international health [3], is grouped into six regions including the African region, which is made up of 47 countries. The African region was the last to be affected by the COVID-19 pandemic with the first case notified on 25 February 2020 in Algeria [4]. By 13 May 2020, after a confirmed case was detected in Lesotho [5], all countries in the WHO African region were affected. As of 3 January 2021, the African region remains one of the least affected regions globally, accounting for 2.4% (*n* = 1.9 million out of 83.3 million) and 2.4% (*n* = 43 600 out of 1.8 million) of globally reported COVID-19 cases and deaths, respectively [6].

Following the notification of the first cases outside China, in Japan on 15 January 2020, countries in the African region started implementing preparedness and readiness measures to set up emergency response systems, increase their capacity to detect, care for patients, communicate on critical risks and prevention measures to all communities and counter misinformation [7]. In March 2020, in addition to public health measures, countries began to implement physical distancing and social measures aimed at slowing down the spread of COVID-19 [8]. These measures included but were not limited to travel restrictions, curfews, school closures and partial or full lockdowns. Such measures were implemented early, in some cases even before countries had detected cases [8]. Between 1 and 19 April 2020, almost all countries in the African region implemented full or partial lockdown measures, which may have resulted in fewer imported cases and reduced intra-country transmission [8, 9]. From late April 2020, some countries started to gradually ease lockdown measures, and at the same time increased their testing capacity. This was followed by an increase in new infections across the region, which reached a peak by the end of July 2020 [10, 11]. From this peak, new cases declined through August and September 2020, and plateauing before increasing again during November and December 2020 [10, 11].

The emergence of new mutant strains of SARS-CoV-2 in the United Kingdom, South Africa and in Brazil and in South Africa [12, 13], both of which appear to be more transmissible, make it even more critical for countries to improve their readiness to respond appropriately to the possibility of a more prolonged resurgence. However, the lockdowns in place across the region between March and May 2020 adversely affected the functioning of health systems and caused significant social and economic disruption, which negatively impacted people's health and wellbeing [7]. As a result, countries are understandably reluctant to impose new nationwide and full lockdowns.

It is, therefore, critical to provide a tool that can be used to assess the risk of overwhelmed healthcare systems as a result of continued spread of the pandemic, not only at national, but also at sub-national levels, in order to inform timely decisions on tailored public health and social measures (PHSM). To this end, the WHO developed new guidance for implementing and adjusting PHSM in the context of the COVID-19 pandemic [14]. This uses an agile decision-making tool to assess the risk of overwhelmed healthcare systems at national and sub-national levels, using a risk/benefit approach that considers the intensity of transmission and the health system's capacity to respond. The objective of this paper was to assess the risk of overwhelmed healthcare systems as a result of continued spread of COVID-19 in the WHO African region using the WHO guidance tool for implementing and adjusting PHSM in the context of the COVID-19 pandemic. We specifically reviewed the status of COVID-19 transmission in each country, assessed the capacity of health systems to respond to an upsurge in COVID-19 cases and estimated the risk of overwhelmed healthcare systems as a result of continued spread of SARS-CoV-2.

## Methods

### Criteria for assessing COVID-19 risk

We carried out the assessment of risk of overwhelmed healthcare systems as a result of continued spread of COVID-19 in all but one of the WHO African region's countries, using both the transmission scenario and the health systems' response capacity in line with the new WHO guidance tool [14]. The risk was defined as the likelihood of occurrence of the disease and the probable magnitude of the consequences of an adverse event during a specified period in a specific area [15].

The WHO has defined seven transmission scenarios to describe the dynamic of the pandemic: no active cases, sporadic cases, clusters of cases and community transmission (CT) with (i) low incidence, (ii) moderate incidence, (iii) high incidence and (iv) very high incidence. Table 1 summarises different transmission scenarios [14].

**Table 1.** Transmission scenarios’ definitions [11]

Transmission scenario	Definition Countries/territories/areas with
No active cases	No new cases detected for at least 28 days (two times the maximum incubation period), in the presence of a robust surveillance system. This implies a near-zero risk of infection for the general population
Sporadic cases	Cases detected in the past 14 days are all imported, sporadic (e.g. laboratory-acquired or zoonotic) or are all linked to imported/sporadic cases, and there are no clear signals of further locally acquired transmission. This implies minimal risk of infection for the general population
Clusters of cases	Cases detected in the past 14 days are predominantly limited to well-defined clusters that are not directly linked to imported cases but, which are all linked by time, geographic location and common exposures. It is assumed that there are a number of unidentified cases in the area. This implies a low risk of infection to others in the wider community if exposure to these clusters is avoided
Community transmission – level 1 (CT1)	Low incidence of locally acquired, widely dispersed cases detected in the past 14 days, with many of the cases not linked to specific clusters; transmission may be focused in certain population sub-groups. Low risk of infection for the general population
Community transmission – level 2 (CT2)	Moderate incidence of locally acquired, widely dispersed cases detected in the past 14 days; transmission less focused in certain population sub-groups Moderate risk of infection for the general population
Community transmission – level 3 (CT3)	High incidence of locally acquired, widely dispersed cases in the past 14 days; transmission widespread and not focused in population sub-groups High risk of infection for the general population
Community transmission – level 4 (CT4)	Very high incidence of locally acquired, widely dispersed cases in the past 14 days Very high risk of infection for the general population

**Fig. 1.**
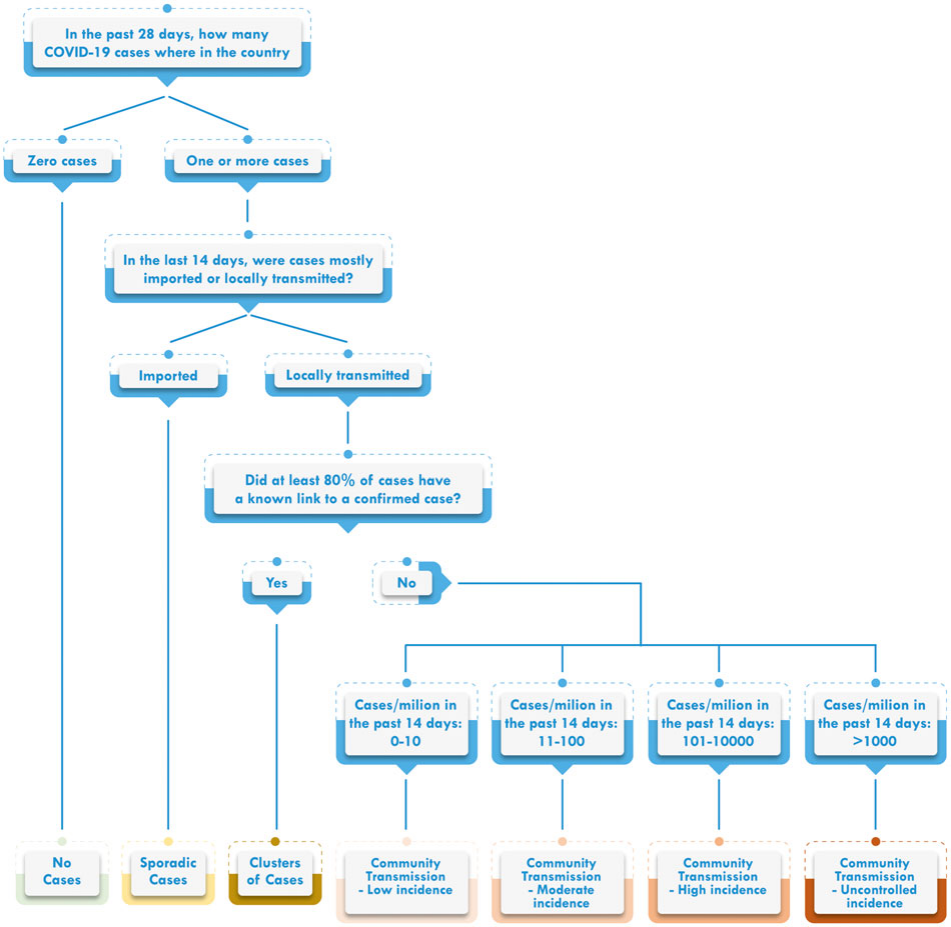
Decision scheme on transmission pattern for countries in the WHO African region.

To assign a transmission scenario to each country, we computed the number of new cases reported in the past 28 days, the proportion of imported cases and locally transmitted cases in the past 14 days and the number of new cases reported in the past 14 days per million population. A decision scheme (Fig. 1) was used to define the transmission scenario of each country.

The capacity of a health system to respond to the COVID-19 pandemic was assessed using the following three indicators: percent of change in new deaths among laboratory-confirmed COVID-19 infections in the past 28 days compared to the previous 28 days, average number of tests per 100 000 population per week during the last 4 weeks and percent change in new health worker infections in the past 28 days compared to the previous 28 days. The range and score of each indicator is summarised in Table 2.

**Table 2.** Indicators and proposed ranges for assessing health system response capacity

No.	Indicator	Score		
		0	1	2
1	Percent of change in new deaths in the past 28 days compared to the previous 28 days	⩽−50	>−50 and <20	⩾20
2	Average number of tests per 100 000 population per week during the last 4 weeks	>200	100–200	<100
3	Percent change in new health worker infections in the past 28 days compared to the previous 28 days	⩽−50	>−50 and <20	⩾20

The overall score for health system's response capacity was derived as the sum of scores for each indicator. The overall performance of the health system was considered as adequate if the overall score ranged between 0 and 2, moderate if it was between 3 and 4 and limited for a score between 5 and 6.

A matrix combining the transmission scenario and health system's response capacity was then used to estimate the level of risk of overwhelmed healthcare systems as a result of continued spread of the pandemic in each country. Four risk levels corresponding to the situational risk level were defined, with level 1 corresponding to a situation with no known transmission of SARS-CoV-2 in the preceding 28 days and level 4 a situation of uncontrolled epidemic with limited or no additional response capacity (Table 3). The detailed interpretation of each risk level and recommended PHSM actions are summarised in Table 4.

**Table 3.** COVID-19 risk for pandemic to continue spreading matrix [11]

Transmission level	Response capacity		
	Adequate	Moderate	Limited
No cases	Level 0	Level 0	Level 1
Imported/sporadic cases	Level 0	Level 1	Level 1
Clusters of cases	Level 1	Level 1	Level 2
CT with low incidence	Level 1	Level 2	Level 2
CT with moderate incidence	Level 2	Level 2	Level 3
CT with high incidence	Level 2	Level 3	Level 3
CT with uncontrolled incidence	Level 3	Level 3	Level 4

**Table 4.** COVID-19 risk levels’ interpretation and actions recommended

Risk level	Corresponding situation	Actions recommended
Level 0	Situation with no known transmission of SARS-CoV-2 in the preceding 28 days	The health system and public health authorities should be ready to respond and there should be no restrictions on daily activities
Level 1	Situation where the epidemic is controlled through effective measures around the cases or clusters of cases, with limited and transient localised disruption to social and economic life	Surveillance should ensure that any new case can be detected and managed as early as possible, but there should be no restrictions on daily activities
Level 2	Situation with low community incidence or a risk of CT beyond clusters	Measures should be applied to limit the number of social encounters in the community while ensuring services can remain open with safety measures in place
Level 3	Situation of CT with limited additional capacity to respond and a risk of health services becoming overwhelmed	Need to strengthen all PHSM to avoid more stringent restrictions on movement and other related measures applied under level 4. All individuals should reduce their social contacts, and some activities may need to close while allowing for essential services, and in particular schools, to remain open
Level 4	Situation of uncontrolled epidemic with limited or no additional response capacity requiring strong measures for control to avoid large excess mortality and for health services to be overwhelmed	More stringent movement restrictions and related measures may need to be put in place to significantly reduce the time-bound and aimed to be as short as reasonably possible

### Inclusion and exclusion criteria

All the 47 countries of the African region that have reported at least one COVID-19 laboratory-confirmed cases to the WHO were considered for this analysis. A laboratory-confirmed case of COVID-19 is defined as any case that was confirmed positive for SARS-CoV-2 genetic material by reverse transcriptase polymerase chain reaction (RT-PCR) test. Countries that did not submit a formal report on new cases and deaths during the 28 days (two maximum incubation periods), including a report of zero cases, were excluded from the transmission scenario analysis, and those that did not share data on the number of tests performed (daily or cumulatively) were excluded from the health system's response capacity and risk analysis.

### Data source and analysis

All data contained in our analysis are based on official COVID-19 data reported to the WHO by the respective Ministries of Health between February and December 2020. These include but are not limited to data on cases and deaths, data on RT-PCR tests performed by each country and indicators used to assess transmission and health system's response capacity. We used R version 4.0.3 [16] for statistical analysis and using ESRI 2017 ArcGIS Pro 2.1.0 [17] for mapping.

## Results

### Transmission scenarios

Between 1 and 31 December 2020, 46 countries out of 47 meeting the study inclusion criteria reported a total of 387 493 confirmed cases and 8875 deaths, giving a case fatality ratio of 2.3%. Tanzania was the only country that did not formally report new cases to the WHO during the period and was, therefore, excluded.

**Fig. 2.**
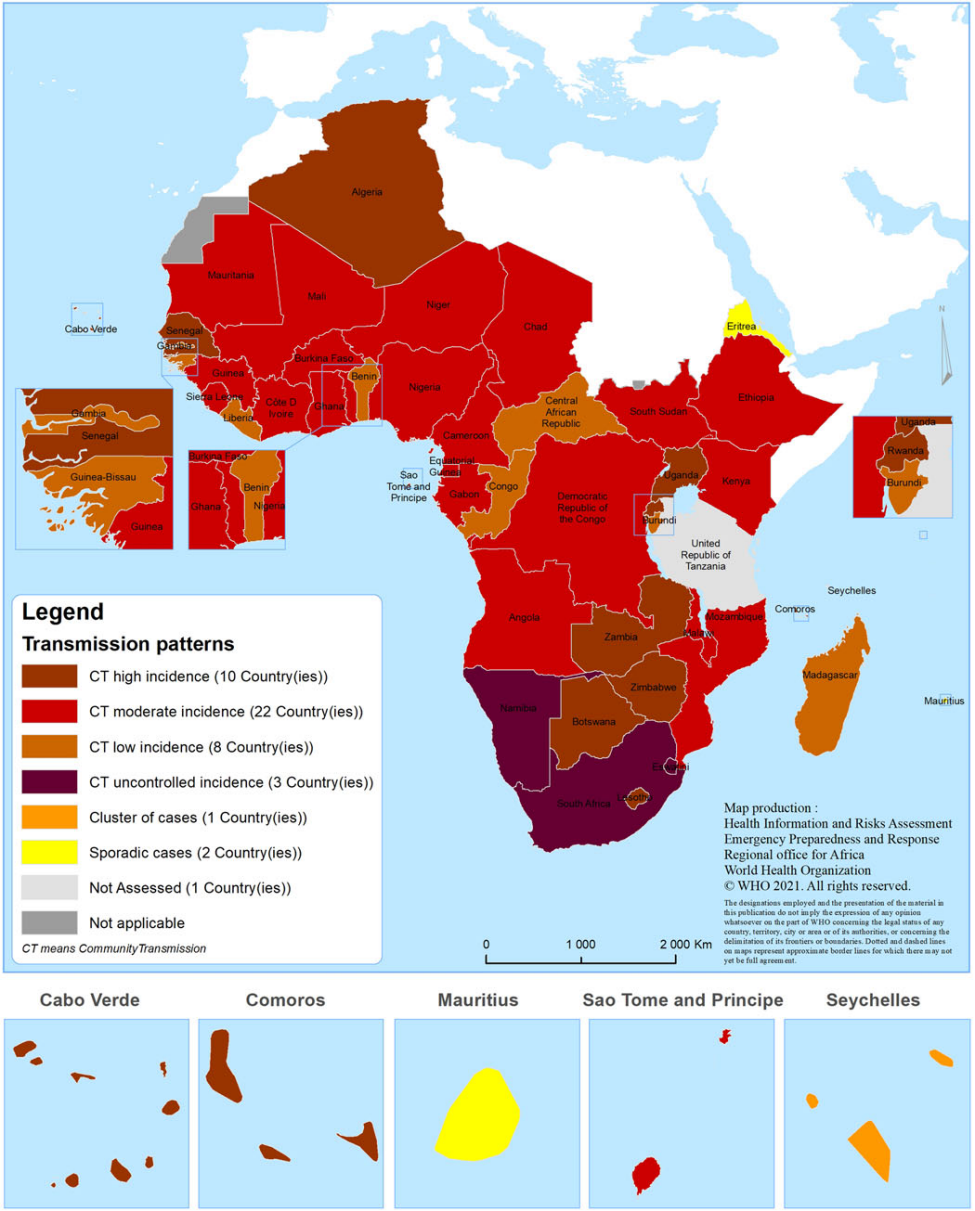
Geographical distribution of countries in the WHO African region by transmission scenario.

Of the 46 countries included, the COVID-19 transmission was classified as sporadic cases in two countries (4.3%), clusters of cases in one country (Seychelles) (2.2%) and CT in the remaining 43 countries (93.5%). Mauritius and Eritrea experienced sporadic transmission of cases and Seychelles experienced clusters of cases. Of the 43 countries experiencing CT, eight experienced CT with low incidence (17.4%), 22 CT with moderate incidence (47.8%), 10 CT with high incidence (21.7%) and three CT with very high incidence (6.5%). The geographical distribution of countries by transmission scenario is illustrated in Figure 2.

### Health system's response capacity

The health system's response capacity was assessed as adequate in two countries (4.3%), moderate in 13 countries (28.3%) and limited in the remaining 31 countries (64.4%).

The percentage of change in the number of COVID-19 deaths recorded in the past 28 days was 20% or above in 40 countries (87.0%), above 50% or less than 20% in five countries (10.9%) and less than 50% in one country (2.1%).

In terms of testing performance, 10 countries performed a weekly average of 20 tests per 100 000 population (21.7%), two countries performed between 10 and 20 tests per 100 000 population (4.3%) and 34 countries performed below 10 tests per 100 000 population (73.9%).

**Fig. 3.**
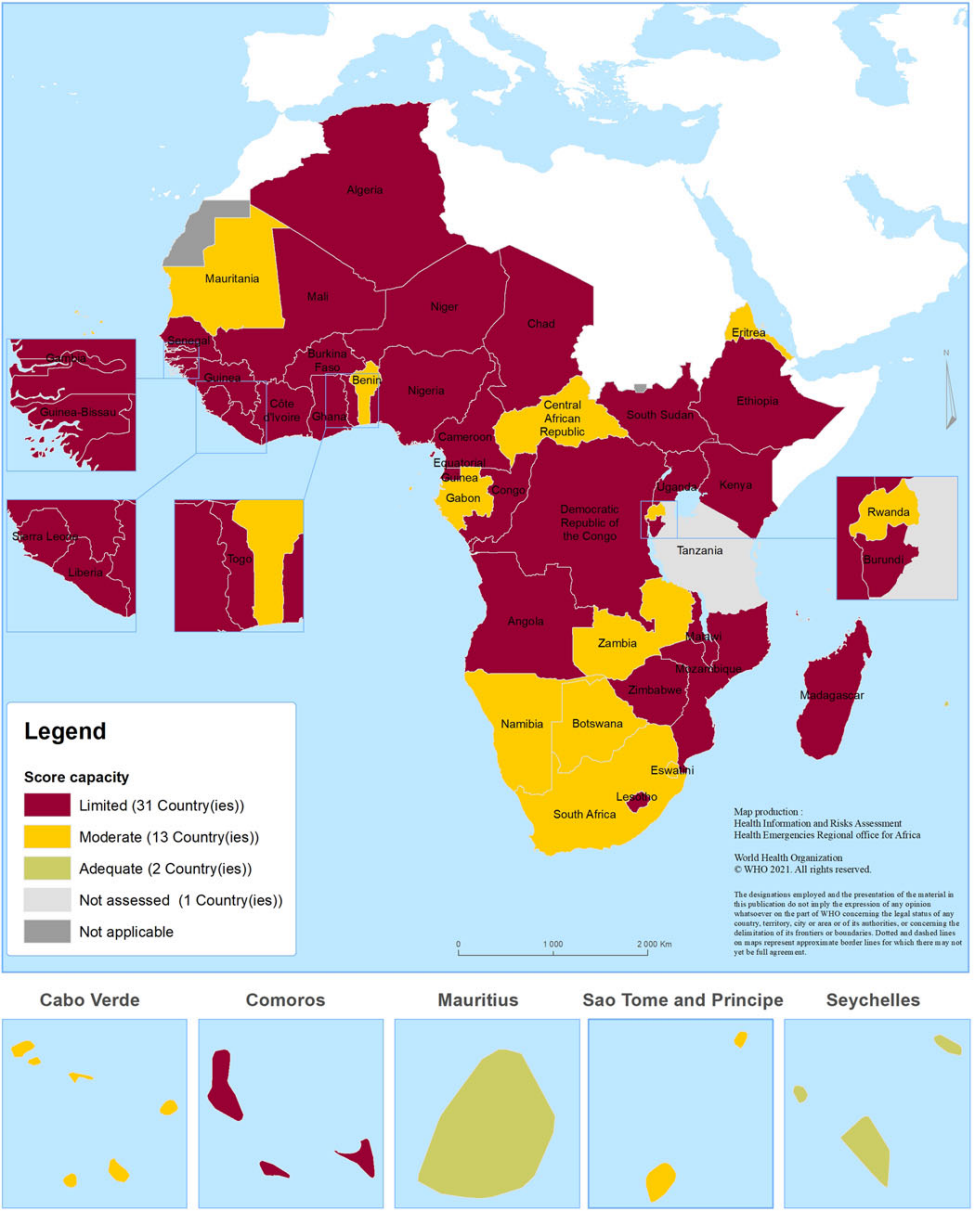
Geographical distribution of countries in the WHO African region by health system's response capacity.

The percentage of change in new health workers’ infections reported in the past 28 days was 20% or above in 10 countries out of 46 assessed (21.7%), above 50% or less than 20% in 35 countries (76.2%) and less than 50% in one country (2.1%) (Figure 3).

### COVID-19 risk level

**Fig. 4.**
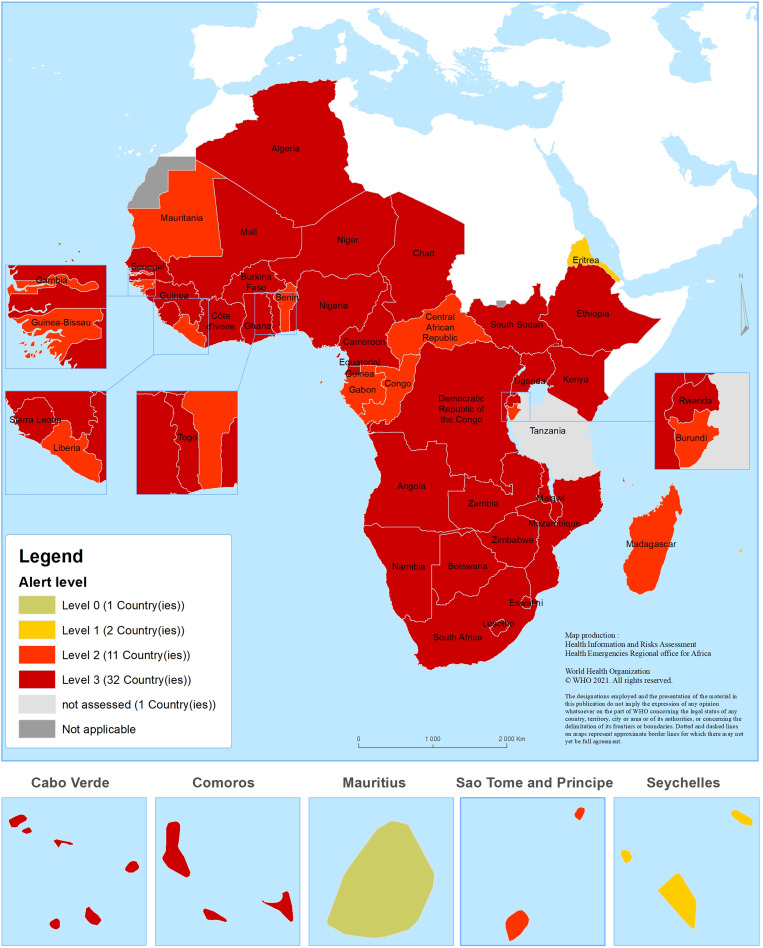
Geographical distribution of countries in the WHO African region by COVID-19 risk level.

The overwhelmed healthcare system as a result of continued spread of COVID-19, using the transmission scenario and the health system response's capacity, was graded at level 0 for one country out of 46 (2.1%), level 1 for two countries (4.3%), level 2 for 11 countries (23.9%) and level 3 for the remaining 32 countries (69.6%). None of the countries assessed met the criteria of level 4. The risk level assigned to each country and the geographical distribution of countries in the WHO African region by COVID-19 risk level are shown in Table 5 and Figure 4, respectively.

**Table 5.** Indicators and reports used to assign a COVID-19 risk level by country in the WHO African region

Country	Cumulative number of cases	New cases in the past 28 days^a^	All cases reported in the last 14 days are imported?	At least 80% of cases reported in the last 14 days are locally transmitted	Epi-link known for at least 80% of cases reported in the last 14 days	Number of cases reported in the past 14 days by million population	% change in new deaths in the past 28 days	Average number of tests per 100 000 population per week during the last 4 weeks^b^	Transmission scenario	Health system's response capacity	Risk level
Algeria	99 610	13 683	No	Yes	No	132	10.1	30	CT high incidence	Limited	level_3
Angola	17 553	2192	No	Yes	No	37	13.5	57	CT moderate incidence	Limited	level_3
Benin	3251	196	No	Yes	No	8	0	29	CT low incidence	Moderate	level_2
Botswana	14 805	3884	No	Yes	No	705	16.7	1427	CT high incidence	Moderate	level_3
Burkina Faso	6828	3737	No	Yes	No	97	20.5	27	CT moderate incidence	Limited	level_3
Burundi	822	130	No	Yes	No	5	50	34	CT low incidence	Limited	level_2
Cameroon	26 848	2096	No	Yes	No	43	1.1	20	CT moderate incidence	Limited	level_3
Cape Verde	11 840	902	No	Yes	No	615	7.1	366	CT high incidence	Moderate	level_3
CAR	4963	36	No	Yes	No	3	0	6	CT low incidence	Moderate	level_2
Chad	2113	405	No	Yes	No	18	2.1	18	CT moderate incidence	Limited	level_3
Comoros	765	150	No	Yes	No	143	22.2	8	CT High incidence	Limited	level_3
Congo	6200	426	No	Yes	No	0	1.1	43	CT low incidence	Limited	level_2
Cote d'Ivoire	22 250	817	No	Yes	No	19	3.6	36	CT moderate incidence	Limited	level_3
DRC	17 658	4377	No	Yes	No	28	17.5	4	CT moderate incidence	Limited	level_3
Eq Guinea	5277	118	No	Yes	No	46	1.2	58	CT moderate incidence	Limited	level_3
Eritrea	1320	726	Yes	Not applicable	Not applicable	180	100	72	Sporadic	Moderate	level_1
Eswatini	9358	2884	No	Yes	Not applicable	1973	43,5	948	CT very high incidence	Moderate	level_3
Ethiopia	124 264	12 685	No	Yes	No	52	11.2	33	CT moderate incidence	Limited	level_3
Gabon	9571	332	No	Yes	No	91	6.5	1274	CT moderate incidence	Moderate	level_2
Gambia	3800	32	No	Yes	No	6	10	48	CT low incidence	Limited	level_2
Ghana	54 930	2656	No	Yes	No	42	27.8	52	CT moderate incidence	Limited	level_3
Guinea	13 738	552	No	Yes	No	18	6.2	41	CT moderate incidence	Limited	level_3
Guinea-Bissau	2447	6	No	Yes	No	0	2.2	21	CT low incidence	Limited	level_2
Kenya	96 458	10 075	No	Yes	No	58	10.8	71	CT moderate incidence	Limited	level_3
Lesotho	2577	432	No	Yes	No	137	4.3	66	CT high incidence	Limited	level_3
Liberia	1800	124	No	Yes	No	4	0	32	CT low incidence	Limited	level_2
Madagascar	17 714	241	No	Yes	No	5	2.3	5	CT low incidence	Limited	level_2
Malawi	6583	540	No	Yes	No	24	3.2	13	CT moderate incidence	Limited	level_3
Mali	7090	2210	No	Yes	No	53	40.8	46	CT moderate incidence	Limited	level_3
Mauritania	13 642	4283	No	Yes	No	28	12.1	217	CT moderate incidence	Moderate	level_2
Mauritius	527	22	Yes	Not applicable	Not applicable	2	0	401	Sporadic	Adequate	level_0
Mozambique	18 642	2724	No	Yes	Not applicable	46	21	30	CT moderate incidence	Limited	level_3
Namibia	24 545	9765	No	Yes	No	2607	21.2	494	CT very high incidence	Moderate	level_3
Niger	3208	1568	No	Yes	No	33	1.9	16	CT moderate incidence	Limited	level_3
Nigeria	87 510	19 207	No	Yes	No	56	8.9	21	CT moderate incidence	Limited	level_3
Rwanda	8383	2372	No	Yes	No	107	45.5	187	CT high incidence	Moderate	level_3
Sao Tome & Principe	1014	17	No	Yes	No	14	0	141	CT moderate incidence	Moderate	level_2
Senegal	19 140	2923	No	Yes	No	104	27	55	CT high incidence	Limited	level_3
Seychelles	267	86	No	Yes	Yes	666	0	4615	Clusters	Adequate	level_1
Sierra Leone	2560	144	No	Yes	No	12	1.3	56	CT moderate incidence	Limited	level_3
South Africa	1 057 561	256 689	No	Yes	No	2813	24.5	473	CT very high incidence	Moderate	level_3
South Sudan	3558	404	No	Yes	No	30	3.2	34	CT moderate incidence	Limited	level_3
Togo	3633	594	No	Yes	No	37	3.4	81	CT moderate incidence	Limited	level_3
Uganda	35 511	13 613	No	Yes	No	123	23	64	CT high incidence	Limited	level_3
Zambia	20 725	2995	No	Yes	No	124	9.3	249	CT high incidence	Moderate	level_3
Zimbabwe	13 867	3443	No	Yes	No	137	24.4	74	CT high incidence	Limited	level_3

CT, community transmission.

^a^Last 28 days (4–31 December 2020).

b Last 14 days (18–31 December 2020).

## Discussion

The response and rapid control of COVID-19 pandemic in the WHO African region depends on the ability and capacity of countries to closely monitor the changes in the COVID-19 transmission pattern, the ability of public health and health system infrastructure to adapt and use by national authorities (decision makers) of PHSM that are informed by scientific data and analysis.

Through the application of WHO guidance on considerations for adjusting PHSM in the context of COVID-19, our study reveals that the majority of countries in the WHO African region were experiencing CT (93.5%, *n* = 43) at the end of 2020, have a health system capacity to respond graded as limited (64.4%, *n* = 31) and a risk of overwhelmed healthcare systems as a result of continued COVID-19 spread at level 3 (69.6%, *n* = 32).

Although at present the WHO African region is the least affected, the high number of countries experiencing CT coupled with the slow vaccination rollout might result in a prolonged outbreak in the region and possible increase in the number of cases and deaths in the coming months [18]. Also, the emergence of more lethal variants of concern due to the persistent circulation of SARS-CoV-2 may make the situation more tragic for African countries

Long-distance truck drivers are an example in Africa, where COVID-19 controls at points of entry increase the time spent at border crossings, which has recently been addressed in the East African sub-region by introduction of specific guidelines to harmonise the strategy for points of entry surveillance, laboratory testing and transnational response to COVID-19 for cross-border truck drivers [19, 20]. This involves a package of infection prevention and control interventions at different stages of their journey.

Although most countries in the region demonstrate CT, the study showed that Eritrea, Seychelles and Mauritius met the criteria of sporadic cases (with all active cases imported) or clusters of cases (with most active cases locally transmitted linked to known confirmed cases or clusters) in the last 28 days of the year 2020. However, in the absence of data from SARS-CoV-2 seroprevalence surveys studies, it is possible that these three countries have experienced CT at some point during the course of the pandemic. In addition, they demonstrated adequate response capacity and remained at risk level 0 or 1. Seychelles and Mauritius are small, isolated island states, with populations of 98 000 and 1.3 million people, respectively [21] and had the ability to close their borders. These two island states, mirroring many other African countries, responded early in the pandemic, mapping fiscal and social protection policy responses to COVID-19 as early as March 2020 [22, 23]. Both countries established treatment and quarantine centres at various locations, as well as setting up response funding, risk communication and community engagement and daily public reports of COVID-19 statistics.

Health system response capacity is a crucial factor in COVID-19, affecting case management, infection prevention and control and overall ability to contain the pandemic. Should case numbers continue to rise, health resource availability will soon be exceeded and the health care workforce, already grossly under supported and ill-equipped, will be exhausted [24]. The high proportion of countries with moderate or limited capacity to respond to an upsurge of COVID-19 cases in the region underscores the urgent need for countries to be supported in strengthening health systems through the implementation of national health policies, strategies and plans, which play an essential role in improving health system capacity and which WHO has recently updated to include specific COVID-19 guidance for COVID-19 response and recovery in fragile settings [25].

The COVID-19 pandemic is the first time since WHO's Emergency Framework was established in 2013 [26] that all 47 Member States have been simultaneously affected by an infectious disease outbreak, challenging the capacity of the WHO Regional Office to provide the required support. Countries with a current risk graded at level 3 should be considered as priority countries for the WHO Regional Office with the aim of preventing the evolution of the situation to level 4, where it may be necessary to enforce hard and costly lockdown measures [27, 28]. The risk assessment tool has been shown to be effective at identifying risk at the national level. However, in order to avoid imposition of potentially costly restrictive measures across the whole country, the tool should also be used at the sub-national level, focusing on local transmission and response capacity, in order to provide a more targeted and localised response.

### Limitations

In this study, we used the change in new cases, health workers infections and the number of tests conducted per 100 000 population reported in the past 28 days to assess the health system's response capacity. These indicators were chosen for the areas of the health system they represent (case management for new cases, health care workforce for health care worker infections, and detection capacity for number of tests conducted per 100 000 population) and considering the available data for most countries of the region. Although adding the hospitalisation rate or intensive care unit beds occupancy would improve the accuracy of this metric, these data points were not as widely available across the region. Furthermore, some countries in the WHO African region are under-reporting health workers infections, which may have resulted in over-estimated health system response capacity. Additionally, delays in reporting new cases and deaths in some countries may have resulted in an under-estimated attack rate over the last 28 days, resulting in a lower CT classification than accurately reflects the current situation. Finally, new cases are determined through testing, so asymptomatic or mild cases who did not seek clinical care or testing are not captured in these metrics. Since most cases have no symptoms, or only mild symptoms, under-detection of COVID-19 cases may not necessarily result in missed deaths to the same extent. These assessments used the best currently available data to guide real-time decision-making, however the interpretation of the results presented here should take these limitations into account.

## Conclusions

Although some countries in the European region are experiencing a second and third wave of the COVID-19 pandemic, the trend in incidence is still declining or plateauing in most countries of the African region, the exception being several Southern Africa countries. Nevertheless, a region-wide resurgence of the pandemic cannot be ruled out. Applying the COVID-19 risk-based approach provided by the WHO to countries of the African region has shown that most countries are at risk level 3, the second-highest level of severity on a scale ranging from 0 to 4. This should serve as a reminder to Member States of the African region of the need to maintain an effective risk management system, adjust response strategy to the pattern of the pandemic and continue to apply physical distancing measures. Building on and learning from the experiences in responding to Ebola virus disease and human immunodeficiency virus, it is critical at the current stage of the pandemic, marked by new infections kept relatively low in the past 2 months and a population fatigue, to have communities as partners for higher buy-in and support of PHSM in every locality. The use of the WHO risk-based approach by Member States at the lowest administrative level can serve as a tool for adjusting and tailoring specific PHSM.

## Data availability statement

The datasets generated during and/or analysed during the current study are available from the corresponding author on a reasonable request.
